# The Calcineurin Inhibitor Tacrolimus Reduces Proteinuria in Membranous Nephropathy Accompanied by a Decrease in Angiopoietin-Like-4

**DOI:** 10.1371/journal.pone.0106164

**Published:** 2014-08-28

**Authors:** Lei Peng, Jing Ma, Rui Cui, Xiao Chen, Shi-Yao Wei, Qiu-Ju Wei, Bing Li

**Affiliations:** 1 Department of Nephrology, 2^nd^ Affiliated Hospital of Harbin Medical University, Harbin, People’s Republic of China; 2 Department of Nephrology, Sichuan Academy of Medical Sciences & Sichuan Provincial People’s Hospital, Chengdu, People’s Republic of China; Center for Interdisciplinary Research in Biology (CIRB) is a novel Collège de France/CNRS/INSERM, France

## Abstract

Tacrolimus is an anticalcineurinic agent with potent immunosuppressive activity that has recently been shown to have the added benefit of reducing proteinuria in membranous nephropathy (MN) patients. However, its potential mechanisms remain unknown. To reveal the mechanism, rat cohorts were administered tacrolimus or vehicle from days 7 to 28 after the induction of passive Heymann nephritis (PHN). PHN induction resulted in heavy proteinuria and increased expression of desmin, a marker of injured podocytes. We also showed that the glomerular expression of angiopoietin-like-4 (Angptl4) was markedly upregulated in PHN rats and human MN followed by an increase in urine Angptl4 excretion. In addition, increased Angptl4 expression may be related to podocyte injury and proteinuria. Furthermore, upregulated Angptl4 expression primarily colocalized with podocytes rather than endothelial or mesangial cells, indicating that podocytes may be the source of Angptl4, which then gradually migrated to the glomerular basement membrane over time. However, tacrolimus treatment markedly reduced glomerular and urinary Angptl4, accompanied by a reduction in the established proteinuria and the promotion of podocyte repair. Additionally, glomerular immune deposits and circulating IgG levels induced by PHN clearly decreased following tacrolimus treatment. In conclusion, this is the first demonstration that the calcineurin inhibitor tacrolimus can reduce Angptl4 in podocytes accompanied by a decrease in established proteinuria and promotion of podocyte repair in MN.

## Introduction

Membranous nephropathy (MN) is one of the leading causes of primary nephrotic syndrome regardless of race, and although spontaneous remissions occur, MN is still an important cause of chronic kidney failure [1], [2]. Circulating autoantibodies that interact with native antigens and embed in the podocyte cell membrane-basement membrane interface are generally regarded as the fundamental pathobiological mechanism of the disease [3]. In situ formation of subepithelial immune deposits alters glomerular capillary permeability through complement-mediated damage of the podocyte and its slit pore membrane. Thus, MN is now regarded as a podocytopathy, which has the following characteristics: subepithelial immune deposits, podocyte foot process effacement and an expanding glomerular basement membrane (GBM). Podocytes play a central role in proteinuria and renal function loss during this process [3].

Therapeutic strategies for MN patients are controversial. The current treatment approach mainly includes immunosuppressive and cytotoxic drugs, and immunosuppressive drugs are the most widely used [4], [5]. Tacrolimus is a macrolide lactone antibiotic with potent immunosuppressive activity. Recent clinical trials showed that tacrolimus was able to induce remission and reduce the risk of worsening renal function in a considerable number of MN patients [6], [7]. However, the mechanism by which tacrolimus acts on MN remains unknown. Tacrolimus has been shown to inhibit T cells and to prevent B cell mitogenesis [8], [9]. This property may partially explain the therapeutic effects of tacrolimus in autoimmune diseases and transplantation [10], [11]. Additionally, other investigations have shown that the podocyte actin cytoskeleton is a direct target of the antiproteinuric effect of the calcineurin inhibitor cyclosporine A (CsA) [12]. Tacrolimus acts on calcineurin, a central signaling controller in eukaryotes [13], and results in multi-systemic side effects, such as hypertension and pathoglycemia [14], [15]. Therefore, exploring the downstream targets of the mechanism by which tacrolimus acts on MN may provide new options for MN clinical therapy.

Angiopoietin-like proteins have been implicated in the development of hypertriglyceridemia [16] and tumor metastasis [17]; additionally, these proteins have functional properties that are different from angiopoietin. Angiopoietin-like-4 (Angptl4) is highly expressed in the liver and adipose tissue, but it is expressed at lower levels in cardiomyocytes, skeletal muscle, and the kidneys [18], [19]. Most circulating Angptl4 in rodents is secreted by the liver as a cleaved protein that binds to high-density lipoprotein particles [20]. Recent research showed that podocyte-secreted glomerular Angptl4 was upregulated in experimental minimal change disease (MCD) and MN, and Angptl4 transgenic rats resulted in a high level of proteinuria, indicating that Angptl4 mediates proteinuria in some types of glomerulonephropathy [21].

To explore the underlying mechanism of tacrolimus in MN, we established passive Heymann nephritis (PHN), a typical animal model of human MN, which imitates the pathological process of human MN [22]. In this study, we demonstrate that the glomerular expression and urine excretion of Angptl4 was significantly increased and may be related to podocyte injury and proteinuria in PHN rats and in human MN. Furthermore, podocytes may be the source of Angptl4, which gradually migrated to the GBM over time. However, tacrolimus treatment markedly reduced Angptl4 expression, and this may be a mechanism by which tacrolimus treatment can decrease proteinuria and improve histological alterations in MN.

## Results

### Tacrolimus reduced proteinuria and ameliorated serum biochemical indicators in PHN rats

**Figure 1:**
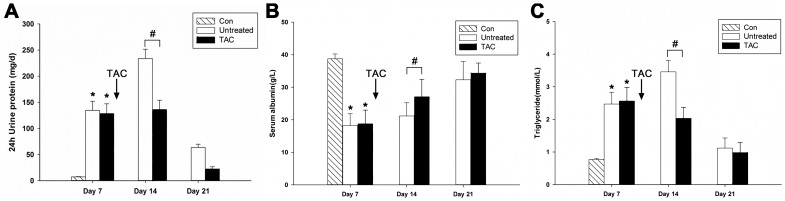
Tacrolimus reduced 24-hour urine protein and ameliorated serum albumin and triglyceride in passive Heymann nephritis (PHN) rats. (**A**) Tacrolimus reduced 24-hour urinary protein excretion in PHN rats. (**B**) Tacrolimus ameliorated serum albumin levels in PHN rats. (**C**) Tacrolimus ameliorated serum triglyceride levels in PHN rats. Con, normal controls; Untreated, PHN rats without treatment; TAC, PHN rats with tacrolimus treatment. Arrows indicate that tacrolimus treatment started on day 7. *P<0.01 vs. normal controls, #P<0.05 vs. PHN. (n = 10 for every group at each time point).

In this study, heavy proteinuria, hypoalbuminemia and hyperlipidemia that are the main clinical features of nephrotic syndrome (including MN) were observed in PHN rats (Fig. 1). Tacrolimus treatment was started on day 7 when heavy proteinuria had been established in PHN rats. There were no differences between the tacrolimus and untreated groups before treatment. After tacrolimus treatment, proteinuria significantly reduced on days 14 and 21 compared with the untreated group (Fig. 1A). In accordance with the markedly reduced proteinuria, serum albumin levels increased (Fig. 1B) and serum triglycerides decreased (Fig. 1C) after tacrolimus treatment. However, there was no significant change in serum cholesterol between the two groups (data not shown).

### Tacrolimus diminished glomerular subepithelial immune deposits and improved foot process effacement in PHN rats

**Figure 2:**
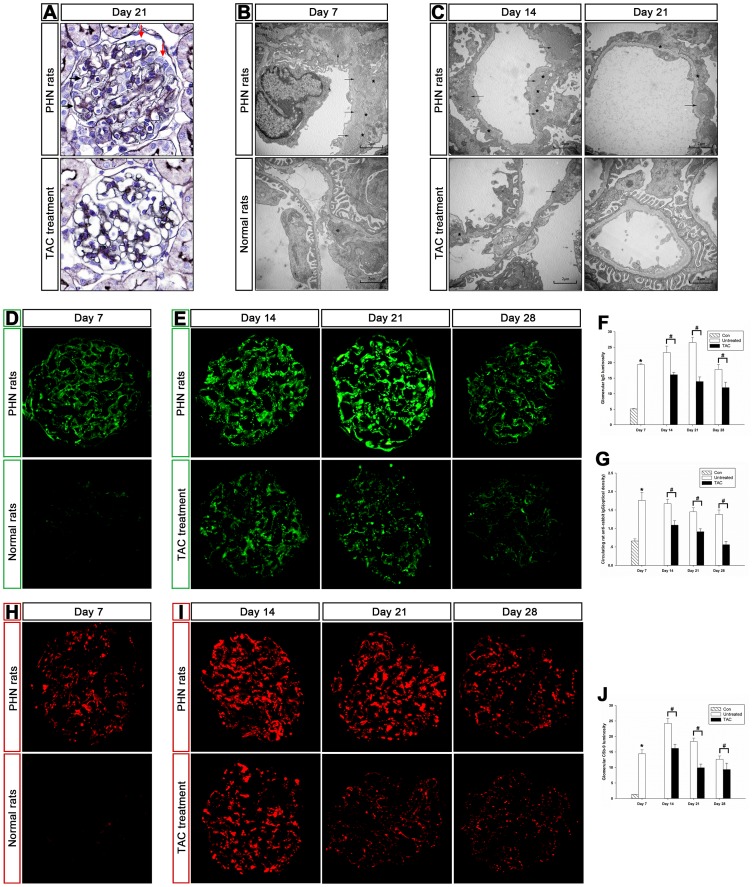
Tacrolimus diminished glomerular subepithelial immune deposits and serum IgG levels and ameliorated foot process effacement in passive Heymann nephritis (PHN) rats. (**A**) PASM staining of tacrolimus-treated and untreated PHN rats on day 21 (magnification, x400). Tacrolimus reversed swollen podocytes to nearly normal shapes and ameliorated the broadened GBM in PHN rats. Black arrows, subepithelial immune depositions. Red arrows, prominent podocytes. (**B and C**) Transmission electron microscopy of normal rats and of tacrolimus-treated and untreated PHN rats. Tacrolimus reduced subepithelial immune deposits and reversed severe foot process effacement and the disappearance of slit diaphragms in PHN rats. Subepithelial immune deposits are indicated with black arrows. Foot process effacement is shown with asterisks. (**D and E**) Immunofluorescence of glomerular rat IgG in normal rats and in tacrolimus-treated and untreated PHN rats (magnification, x400). (**F**) Quantification of the intensity of fluorescent staining for glomerular rat IgG. *P<0.01 vs. normal controls; #P<0.01 vs. PHN. Tacrolimus markedly reduced glomerular IgG levels. (**G**) Circulating rat anti-rabbit IgG antibodies. *P<0.01 vs. normal controls; #P<0.01 vs. PHN. Tacrolimus markedly reduced serum IgG levels. (**H and I**) Immunofluorescence of glomerular C5b-9 in normal rats and in tacrolimus-treated and untreated PHN rats (magnification, x400). (**J**) Quantification of the fluorescent staining intensity of glomerular C5b-9. *P<0.01 vs. normal controls; #P<0.01 vs. PHN. Tacrolimus markedly reduced glomerular C5b-9 deposits.

Glomerular subepithelial immune deposits and foot process effacement are the predominant pathological features of MN [3]. Tacrolimus significantly reduced subepithelial immune deposits and reversed foot process effacement in PHN rats (Fig. 2). On day 7 of PHN, the irregularly broadened GBM and subepithelial immune deposits could be observed by periodic acid-silver methenamine (PASM) staining (data not shown) and electron microscopy (Fig. 2B). Tacrolimus reduced subepithelial immune deposits on days 14 and 21, and the GBM became almost linear as in normal rats on day 21 (Fig. 2A and C).

Tacrolimus markedly reduced serum and glomerular IgG levels (Fig. 2D–G) as well as glomerular C5b-9 deposits (Fig. 2H–J) in PHN rats. Immunofluorescence demonstrated that glomerular IgG and C5b-9 deposition was significantly increased in PHN rats in granular and dispersive patterns along the capillaries compared with normal rats (Fig. 2D and H). The intensity of glomerular IgG and C5b-9 deposition decreased after treatment on days 14, 21, and 28 (Fig. 2E and I). This result is also expressed graphically in Fig. 2F and J. In accordance with glomerular IgG deposition, circulating IgG levels decreased after tacrolimus treatment (Fig. 2G).

Tacrolimus reversed swollen podocytes to nearly normal shapes in PHN rats on day 21 as determined by PASM staining (Fig. 2A) and reversed severe foot process effacement and the disappearance of slit diaphragms in PHN rats on days 14 and 21 as determined by electron microscopy (Fig. 2B and C). These findings indicate that tacrolimus markedly reversed podocyte injury in PHN rats.

### Tacrolimus diminished Angptl4 in podocytes, and Angptl4 gradually migrated to the GBM in PHN rats

**Figure 3:**
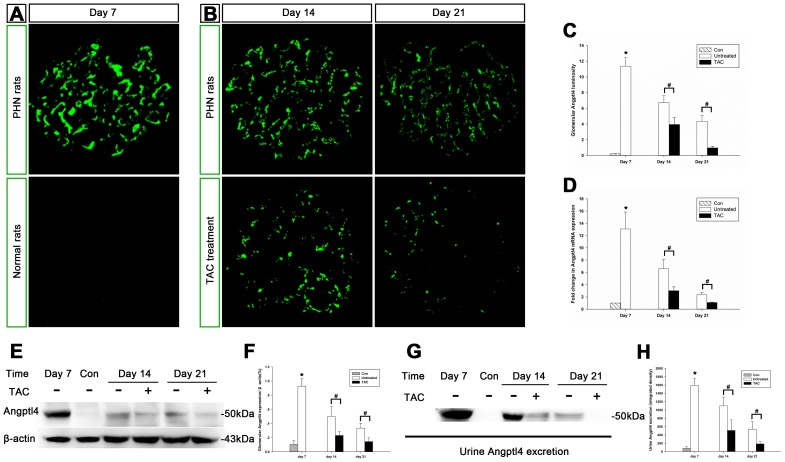
Tacrolimus diminished glomerular Angptl4 in passive Heymann nephritis (PHN) rats. (**A and B**) Immunofluorescence of glomerular Angptl4 in normal rats and in tacrolimus-treated and untreated PHN rats (magnification, x400). (**C**) Quantification of the fluorescent staining intensity of glomerular Angptl4. *P<0.01 vs. normal controls; #P<0.01 vs. PHN rats. (**D**) Quantitative real-time PCR of Angptl4 from kidney tissue. *P<0.01 vs. normal controls; #P<0.05 vs. PHN rats. (**E**) Western blot analysis of glomerular Angptl4 expression in PHN rats. (**F**) Quantification of the western blot analysis of glomerular Angptl4 expression. *P<0.01 vs. normal controls; #P<0.05 vs. PHN rats. Glomerular Angptl4 expression was upregulated in PHN rats and tacrolimus notably diminished glomerular Angptl4. (**G**) Western blot analysis of urine Angptl4 excretion in PHN rats. (**H**) Quantification of the western blot analysis of urine Angptl4 excretion in PHN rats. *P<0.01 vs. normal controls; #P<0.05 vs. PHN rats. Urine Angptl4 excretion was upregulated in PHN rats and tacrolimus notably diminished urine Angptl4. (n = 10 for every group at each time point).

Recently, some evidence has shown that Angptl4 is involved in proteinuria in nephrotic syndrome [21]. We noted that glomerular Angptl4 expression was upregulated in PHN rats on days 7, 14, and 21 compared with normal rats as determined by immunofluorescence (Fig. 3A–C). This finding was also confirmed by quantitative real-time PCR (Fig. 3D) and western blot analysis (Fig. 3E and F). Additionally, increased Angptl4 was excreted into the urine (Fig. 3G and H). The intensity of glomerular Angptl4 was the most significant on day 7, with an agglomerate pattern, and gradually decreased on days 14 and 21 in granular and disperse patterns along the capillary walls in PHN rats (Fig. 3A and B). Tacrolimus notably diminished glomerular Angptl4 (Fig. 3B–F) as well as urine Angptl4 excretion (Fig. 3G and H) on days 14 and 21.

To determine the source of urine Angptl4 excretion, we studied Angptl4 expression in the livers of PHN rats on day 7 using western blot and reverse-transcription PCR. Angptl4 expression in the liver was almost the same between the normal rats and PHN rats (Figure S1), indicating that the liver was unlikely to be the source of urine Angptl4 excretion.

We used a normal control and two types of negative controls for immunofluorescence in PHN rats and diabetic rats to exclude the non-specificity of this Angptl4 antibody in the kidney tissue. The result was shown in Figure S2.

**Figure 4:**
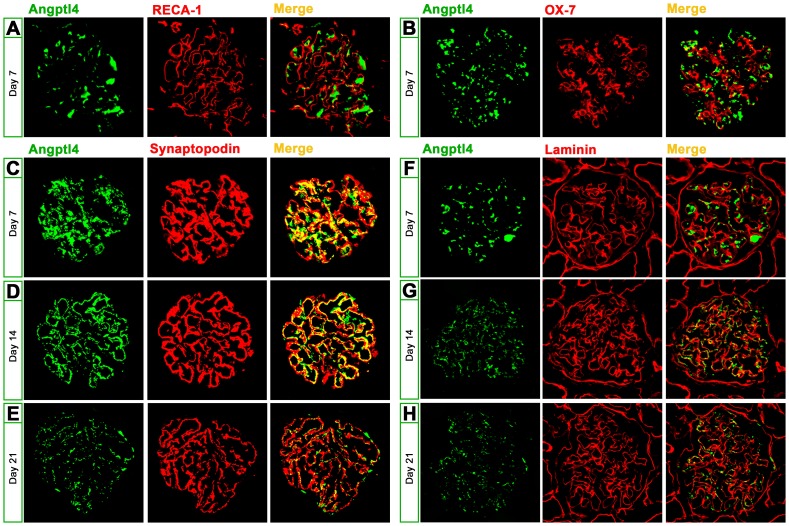
Angptl4 in podocytes gradually migrated to the GBM in passive Heymann nephritis (PHN) rats (magnification, x400). (**A**) Immunofluorescence of glomerular Angptl4 with RECA-1, an endothelial marker, in PHN rats on day 7. (**B**) Immunofluorescence of glomerular Angptl4 with OX-7, a mesangial cell marker, in PHN rats on day 7. (**C–E**) Immunofluorescence of glomerular Angptl4 with synaptopodin, a podocyte marker, in PHN rats on days 7, 14, and 21. The overlap rates of Angptl4 and synaptopodin on days 7, 14, and 21 were 71.16%, 71.58%, and 56.23%, respectively. (**F–H**) Immunofluorescence of glomerular Angptl4 with laminin, a GBM marker, in PHN rats on days 7, 14, and 21. The overlap rates of Angptl4 and laminin on days 7, 14, and 21 were 17.69%, 35.37%, and 29.75%, respectively.

To determine the type of glomerular cell that secretes Angptl4 in PHN rats, we co-stained glomerular Angptl4 with RECA-1 (an endothelial marker), OX-7 (a mesangial cell marker), synaptopodin (a podocyte marker), and laminin (a GBM marker) in PHN rats. Glomerular Angptl4 expression in PHN rats was separate from the endothelium (Fig. 4A) and mesangial cell markers (Fig. 4B) on day 7; however, Angptl4 effectively colocalized with the podocyte marker on day 7 (71.16% of Angptl4 was coincident with synaptopodin, Fig. 4C) and on day 14 (71.58% of Angptl4 was co-localized with synaptopodin, Fig. 4D), indicating that glomerular Angptl4 was most likely secreted by podocytes. Glomerular Angptl4 had gradually separated from podocyte protein synaptopodin in PHN rats on day 21 (56.23% of Angptl4 was co-localized with synaptopodin, Fig. 4E), indicating that Angptl4 in podocytes migrated outside the podocyte. Furthermore, Angptl4 co-staining with laminin showed that glomerular Angptl4 rarely localized with this GBM marker in PHN rats on day 7 (17.69% Angptl4 co-localized with laminin, Fig. 4F). However, the co-localization of glomerular Angptl4 and laminin increased over time to 35.37% on day 14 (Fig. 4G) and 29.75% on day 21 (Fig. 4H), indicating that Angptl4 in podocytes gradually migrated out of the podocytes to the GBM in PHN rats.

### Tacrolimus promoted podocyte repair, and increased Angptl4 expression may be related to podocyte injury and proteinuria in PHN rats

**Figure 5:**
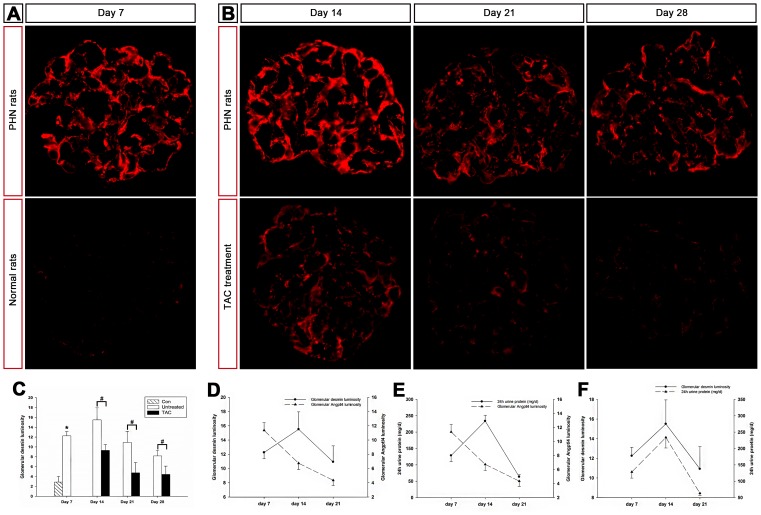
Tacrolimus reduced the expression of glomerular desmin, a biomarker of podocyte injury, and the relationship between Angptl4, desmin, and urine protein. (**A and B**) Immunofluorescence of glomerular desmin in normal rats and in tacrolimus-treated and untreated PHN rats (magnification, x400). (**C**) Quantification of the fluorescent staining intensity of glomerular desmin. *P<0.01 vs. normal controls; #P<0.05 vs. PHN. Tacrolimus reduced the expression of glomerular desmin. (**D**) Relationship between Angptl4 and desmin in PHN rats. The expression of Angptl4 was upregulated to its peak prior to desmin expression. (**E**) Relationship between Angptl4 and urine protein in PHN rats. The expression of Angptl4 was upregulated to its peak prior to proteinuria. (**F**) Relationship between desmin and urine protein in PHN rats. Podocyte injury and proteinuria had the same trends.

Podocytes play a central role in proteinuria development in glomerular diseases [3]. We investigated whether tacrolimus could promote podocyte repair after injury by evaluating the expression of desmin, a biomarker for injured podocytes. Our data indicated that desmin expression was significantly upregulated in PHN rats relative to normal rats (Fig. 5A). Desmin expression intensity after tacrolimus treatment was significantly lower compared with the untreated group on days 14, 21, and 28 (Fig. 5B; shown graphically in Fig. 5C). These data are consistent with the histological alterations observed under light and electron microscopy (Fig. 2A–C).

To determine the relationships between podocyte injury, Angptl4 and proteinuria, we created line charts of Angptl4 expression, desmin expression and proteinuria levels in PHN rats from day 7 to 21. Podocyte injury and proteinuria exhibited the same trends (Fig. 5F), and the expression of Angptl4 was upregulated to its peak prior to desmin expression and proteinuria (Fig. 5D and E), indicating that Angptl4 in podocytes was most likely related to podocyte injury and proteinuria in PHN rats.

### Angptl4 expression in human glomerulonephritis and its relationship to podocyte injury and proteinuria

**Figure 6:**
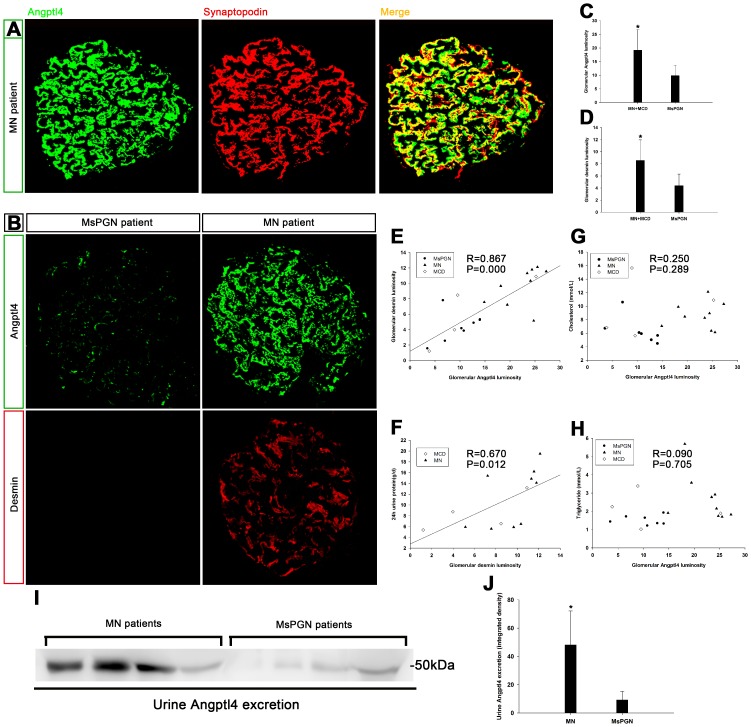
Expression of Angptl4 in human glomerulonephritis and its relationship with desmin and urine protein. (**A**) Immunofluorescence of glomerular Angptl4 with synaptopodin in MN patients (magnification, x400). The overlap rate of Angptl4 and synaptopodin was 58.72%. (**B**) Immunofluorescence of glomerular Angptl4 and desmin in MN and MsPGN patients with similar nephrotic-range proteinuria (magnification, x400). Angptl4 and desmin expression levels were markedly upregulated in the MN patient compared with the MsPGN patient. (**C and D**) Quantification of the immunofluorescence intensities of glomerular Angptl4 and desmin in MN and MCD and non-podocytopathy (MsPGN). *P<0.01 vs. MsPGN. The expression levels of Angptl4 and desmin were notably increased in MN and MCD patients relative to MsPGN patients. (**E**) Scatter diagram of Angptl4 and desmin in 20 patients. A correlative analysis revealed positive correlations between glomerular Angptl4 and desmin expression. (**F**) Scatter diagram of desmin and urine protein in podocytopathy patients (13 patients). Correlative analysis revealed positive correlations between desmin and proteinuria. (**G and H**) The scatter diagrams reveal the relationships between Angptl4, cholesterol, and triglycerides in 20 patients. There were no correlations between glomerular Angptl4 expression and cholesterol or between Angptl4 and triglycerides. (**I**) Western blot analysis of urine Angptl4 excretion in MN and MsPGN patients with similar nephrotic-range proteinuria. (**J**) Quantification of the western blot analysis of urine Angptl4 excretion in MN and MsPGN patients. *P<0.05 vs. MsPGN. Urine Angptl4 excretion was more obvious in MN patients than in MsPGN patients.

To further study Angptl4 expression in glomerulonephritis, we stained Angptl4 and desmin in samples from 20 patients with different types of glomerulonephritis; their baseline characteristics are described in Table 1. In two patients with similar nephrotic-range proteinuria (MN6 and mesangial proliferative glomerulonephritis (MsPGN) 2), Angptl4 and desmin expression levels were markedly upregulated in the MN patient compared with the MsPGN patient (Fig. 6B). In 4 MN patients and 4 MsPGN patients with similar proteinuria (MN1, 2, 5, and 7; MsPGN1, 4, 6, and 7), urine Angptl4 excretion was more obvious in MN patients than in MsPGN patients (Fig. 6I and J). The above results indicate that Angptl4 expression was likely related to MN. Immunofluorescence demonstrated that the expression levels of Angptl4 and desmin were notably increased in MN and MCD patients relative to non-podocytopathy patients (MsPGN patients, Fig. 6C and D), confirming that the enhanced Angptl4 expression was most likely associated with podocyte injury. A correlative analysis revealed positive correlations between glomerular Angptl4 and desmin expression in all 20 patients (R = 0.867, P<0.001, Fig. 6E) and between desmin and proteinuria in MN and MCD patients (R = 0.670, P = 0.012, Fig. 6F). In MN patients, 58.72% of the Angptl4 was coincident with synaptopodin (Fig. 6A), indicating that the increased Angptl4 might be secreted by podocytes in MN patients. There were no correlations between glomerular Angptl4 expression and cholesterol (R = 0.250, P = 0.289, Fig. 6G) or between Angptl4 and triglyceride in all patients (R = 0.090, P = 0.705, Fig. 6H), indicating that Angptl4 in podocytes may primarily work locally in glomeruli instead of through a systemic function.

**Table 1:** Baseline characteristics of the enrolled patients.

Patient	Age (years)	Gender	24 h Urine Protein (g/d)	Serum Albumin (g/L)	Serum Creatinine (µmol/L)
MN1	60	M	5.59	24.8	73
MN2	57	M	5.92	27.3	60
MN3	48	M	14.11	23.1	67
MN4	46	M	14.87	21.5	59.9
MN5	18	F	6.48	26.1	54
MN6	48	M	19.5	19.4	55.8
MN7	38	F	5.89	25.1	72.7
MN8	62	F	15.41	21.8	57.1
MN9	49	M	16.21	20.5	50.5
MCD1	56	M	6.54	42.4	86.9
MCD2	29	F	13.2	16.9	44.3
MCD3	21	M	8.75	20.3	99
MCD4	56	F	5.37	19.1	63.5
MsPGN1	28	M	3.8	30.6	73
MsPGN2	52	M	17.8	10.1	115.8
MsPGN3	27	M	1.8	34.6	104.2
MsPGN4	22	M	3.9	46.4	82
MsPGN5	36	F	1.9	46.2	103.4
MsPGN6	30	F	4.3	33.2	211.4
MsPGN7	42	F	3.7	41.9	55.6

M, male; F, female.

### Adverse effects of tacrolimus treatment

To monitor adverse effects, blood glucose, serum creatinine (Scr) levels, and systolic blood pressure (SBP) were examined. No obvious adverse effects were observed after oral tacrolimus administration at a dose of 1 mg/kg per day (Table 2).

**Table 2:** Adverse effects of tacrolimus treatment in PHN rats on day 28.

Groups	Serum Creatinine (µmol/L )	Blood Glucose (mmol/L )	Systolic Blood Pressure (mmHg)
Tacrolimus treatment	27.5±3.33	8.21±0.79	130.00±8.12
Untreated	29.03±3.8	8.29±0.90	121.25±8.54

## Discussion

In this study, we demonstrated that glomerular and urinary Angptl4 was significantly increased and was most likely associated with podocyte injury and heavy proteinuria in both PHN rats and MN and MCD patients. Furthermore, the upregulated Angptl4 expression was most likely due to podocyte secretion and Angptl4 gradually migrated toward the GBM over time. However, tacrolimus markedly reduced Angptl4 expression and excretion, which was accompanied by a reduction in the established proteinuria and the promotion of podocyte repair in PHN rats. Additionally, the glomerular immune deposits and circulating IgG levels induced by PHN clearly decreased after tacrolimus treatment. Therefore, this study is the first demonstration that in addition to its immunosuppressive function in PHN, tacrolimus may act on Angptl4 in podocytes to reduce proteinuria in MN. However, we cannot definitively prove the relationship between Angptl4 and podocyte injury and cannot exclude the direct effect of tacrolimus on podocytes.

Tacrolimus has been shown to increase the remission rate of proteinuria and reduce the risk of renal function worsening compared with other therapies [6], [7]. The immunosuppressive effects of tacrolimus [8], [9] may partially explain its therapeutic effects in autoimmune diseases and transplantation [10], [11]. In addition, calcineurin inhibitors can also reduce proteinuria in Alport syndrome, which is a non-immunological disease [23]. Other investigations have found that the actin cytoskeleton of podocytes is a direct target of the antiproteinuric effect of the calcineurin inhibitor CsA [12]. Therefore, we hypothesized that tacrolimus may have the added benefit of anti-proteinuria activity in MN independent of its immunological effects.

We have demonstrated that tacrolimus can significantly suppress the production of circulating rat IgG antibodies, decrease subepithelial immune deposits and reduce the expression of glomerular IgG and C5b-9 in PHN rats. Therefore, the effect of tacrolimus on the immune system may also partly reduce proteinuria in PHN rats. However, a recent clinical trial revealed that tacrolimus could induce remission of nephrotic MN much earlier than cyclophosphamide (CTX) [7], which is also an immunosuppressive agent, indicating that there may be other targets of the antiproteinuric effects of tacrolimus aside from its immunosuppressive function in the early phase of MN.

Recent studies on secreted Angptl4 focused mainly on lipid metabolism, tumor metastasis and cardiovascular or cerebrovascular diseases. Previously, Angptl4 has been identified as a regulator of lipid metabolism [24] and as a target of peroxisome proliferator-activated receptors (PPARs) [18]. However, a recent study found that the glomerular expression of Angptl4 was linked to proteinuria and highly increased in serum and podocytes in experimental models of MCD and in human MCD [21]. Angptl4 is expressed in numerous cell types, including adipocytes and hepatocytes, and increases after fasting and hypoxia [18], [25], whereas there was almost no Angptl4 expression in normal rat kidneys in the present study.

In this study, we found that glomerular Angptl4 expression was clearly upregulated in PHN. Angptl4 expression had the highest level on day 7 with an agglomerate pattern and gradually decreased on days 14 and 21 with granular and disperse patterns along the capillary walls in PHN rats, implying that Angptl4 was secreted by some type of renal parenchymal cell in an early phase of PHN and then gradually migrated to another location. Furthermore, double immunofluorescence demonstrated that podocytes might be the source of the additional Angptl4 (with an approximate 71% overlap with podocytes). A similar result was also confirmed in human kidney tissues. We also found that Angptl4 in podocytes gradually migrated to the GBM, indicating that Angptl4 in podocytes may induce a filtration defect in the GBM. A previous study [21] reported that almost all Angptl4 expression co-localized with podocytes on day 6 in the puromycin aminonucleoside model. In the present study, we measured only 71% Angptl4 overlap with podocytes. One possible explanation may be the use of different animal models and different time points. We noticed that after podocyte secretion, Angptl4 moved toward the GBM rather than remaining within the podocytes; this finding is similar to a recent study showing that Angptl4 was localized in podocyte foot processes, in the GBM and close to the endothelial cell surface [21]. Interestingly, Angptl4 can also be detected in the urine of PHN rats and the urine Angptl4 excretion exhibited a tendency analogous to that of glomerular Angptl4 expression. Western blot and reverse-transcription PCR analysis of Angptl4 in the liver of PHN rats on day 7 showed that the liver was unlikely to be the source of urine Angptl4 excretion. The urine Angptl4 excretion most likely came from increased Angptl4 expression in glomeruli. Furthermore, tacrolimus significantly reduced glomerular Angptl4 expression and urine Angptl4 excretion in PHN rats. To our knowledge, this study is the first to demonstrate that tacrolimus decreases Angptl4 expression, which may lead to a reduction in proteinuria.

Our study proved only indirectly the causality between the expression of Angptl4, podocyte injury and proteinuria by determining the sequence of their peaks using line charts; however, Clement LC et al. [21] proved the causality directly. They established podocyte-specific Angptl4 transgenic mice and rats, and found that the urine protein to creatinine ratio increased in these transgenic mice compared with wild-type mice, indicating that podocyte-secreted Angptl4 resulted in proteinuria. Diffuse foot process effacement emerged in podocyte-specific Angptl4 transgenic rats, indicating that podocyte-secreted Angptl4 resulted in podocyte injury.

There is a considerable amount of data emphasizing the pivotal role of podocytes in proteinuria in many different forms of glomerular disease [26]. The calcineurin inhibitor CsA has a direct effect on podocytes [12]. As a calcineurin inhibitor, we expected that tacrolimus also had a direct effect on podocytes. Enhanced desmin expression and extensive podocyte foot process effacement were observed in PHN rats. However, tacrolimus treatment significantly reduced the increased desmin expression and reversed the foot process effacement, indicating that tacrolimus promotes podocyte repair in PHN rats. According to other studies, the possible mechanism could involve promoting podocyte cytoskeleton stabilization [12], [27]. Furthermore, we found positive correlations between Angptl4 and podocyte injury and between podocyte injury and proteinuria in human MN, MCD and MsPGN. Additionally, the degree of podocyte injury exhibited the same trend as proteinuria, and Angptl4 expression peaked prior to desmin expression and urine protein excretion in PHN rats, implying that Angptl4 in podocytes may cause podocyte injury and increased proteinuria. In human glomerulonephritis, glomerular Angptl4 expression and urine Angptl4 excretion were clearly increased in MN and MCD patients but not in non-podocytopathy MsPGN patients, indicating that Angptl4 may be related to podocyte injury in humans. The correlative analysis between increased glomerular Angptl4 and blood lipids showed that Angptl4 may primarily work locally in glomeruli instead of affecting systemic functions.

The upstream mechanisms that cause increased Angptl4 in podocytes are not known and should be explored further. We cannot exclude the immunosuppressive properties and direct effect of tacrolimus on the glomerular filtration barrier. This area requires further research. Future studies will determine whether tacrolimus can reduce Angptl4 expression and proteinuria in any other non-immunological experimental models with massive proteinuria. Because Angptl4 can be detected in the urine of MN patients and glomerular Angptl4 expression may be related to podocyte injury, further studies are needed to explore whether urine Angptl4 excretion can be a non-invasive maker to indicate podocyte injury.

In conclusion, this study is the first demonstration that in addition to its immunosuppressive function in PHN, tacrolimus may act on Angptl4 in podocytes to reduce proteinuria in MN. However, we cannot definitively prove the relationship between Angptl4 and podocyte injury and cannot exclude the direct effect of tacrolimus on podocytes. These questions require further study in the future.

## Materials and Methods

### Ethics statement

Animal experiments were performed in strict accordance with the National Institutes of Health Guidelines for the Care and Use of Laboratory Animals. The Animal Experiments Committee of Harbin Medical University approved all animal care and experimental procedures. Rats were sacrificed under anesthesia (10% chloral hydrate, peritoneal injection), and all efforts were made to minimize pain and discomfort. We conducted our human subject research with the approval of the institutional review boards of the 2nd Affiliated Hospital of Harbin Medical University in Harbin, China. All of the participants provided their written informed consent according to the latest version of the Helsinki Declaration of human research ethics.

### Induction of PHN

The PHN model is a well-characterized rat analog of human MN. PHN was induced following a standard protocol [28]. Fx I A antigen in the brush border was extracted from the renal cortices of Wistar rats. Male New Zealand white rabbits were immunized with emulsive Fx I A antigen, and rabbit antiserum was prepared. PHN was induced in adult male Sprague-Dawley (SD) rats (Harbin Medical University 2nd Affiliated Hospital Laboratories) with body weights of 180 to 200 grams through a single intraperitoneal injection of anti-Fx I A antiserum (n = 70), and preimmune rabbit serum (n = 10) served as a normal control (7 ml/kg body wt). Thirty PHN rats received orally-administered tacrolimus (Astellas, County Kerry, Ireland; 1 mg/kg/d) from day 7 after antiserum injection when proteinuria was already present until sacrifice and were randomly divided into three time point groups: day 14, day 21 and day 28 (n = 10 for each group). Another 40 PHN control rats were administered an oral dose of normal saline and were randomly divided into four time point groups: day 7, day 14, day 21, and day 28 (n = 10 for each group).

### Sample collection and serum parameter measurements

PHN rats were housed in an air-conditioned room and were given free access to food and water (22±2°C; 12∶12-hour light-dark cycle). From PHN induction until sacrifice, 24-hour urine was collected weekly from individual rats in metabolic cages with free access to water but without food. The urine protein content was determined using the nephelometry method (Siemens BN II, Deerfield, IL, USA). On days 7, 14, 21 and 28 after PHN induction, 10 rats from each group were sacrificed under anesthesia. Blood samples were taken from the retro bulbar plexus in anesthetized rats and were immediately analyzed in an automatic biochemistry analyzer (Roche, Cobas c 311, Mannheim, Germany) to measure serum levels of albumin, triglycerides and creatinine, as previously reported [29]. Systolic blood pressure (SBP) was measured using a tail cuff according to the manufacturer’s instructions (Softron BP-98A, Tokyo, Japan). Measurements were obtained in each conscious rat immediately before sacrifice in all groups. The rats were pre-warmed to 36°C for 15–20 minutes in a bag before each measurement. The average of three pressure readings was recorded for each measurement. Renal tissues were prepared for morphological studies, immunofluorescence microscopy, and molecular biology experiments.

Human kidney tissues were collected during renal biopsy from the Nephrology Department in the 2nd Affiliated Hospital of Harbin Medical University and were processed for immunofluorescent staining, as described below. Human urine was collected for 24 hours before treatment.

### Histological studies by light microscopy

The tissue used for light microscopy was fixed in 10% neutral-buffered formalin for 12 hours, dehydrated in graded ethanol, embedded in paraffin for sectioning (2 µm) and stained with hematoxylin-eosin (HE), periodic acid-Schiff (PAS), PASM and Masson’s trichrome. Images were captured using a Nikon DS Ri1 (Tokyo, Japan).

### Morphological studies by transmission electron microscopy

Renal cortex tissue (1 mm^3^) was fixed in cold 2.5% glutaraldehyde for 4 hours. After washing three times in 1 M phosphate buffer (pH 7.2), the renal tissue was fixed in 1% osmium tetroxide for 2 hours. The tissues were washed in phosphate buffer, dehydrated in graded acetone and ethanol and embedded. Ultra-thin sections (80–90 nm) were stained with uranyl acetate and lead citrate and then examined and photographed using a Hitachi 7650 transmission electron microscope (Tokyo, Japan).

### Enzyme-linked immunosorbent assay (ELISA)

The concentrations of rat serum antibodies to rabbit IgG were determined by ELISA. Plates (NUNC, New York, NY, USA) were coated with coating buffer (4.0 mg/ml rabbit IgG in 0.1 M sodium carbonate buffer (pH 9.6)) and incubated for 20 hours at 4°C. The plate was blocked with 1% bovine serum albumin (BSA) for 1 hour at room temperature and then washed with phosphate-buffered saline with Tween 20 (PBST) (10 mM PBS, pH 7.4, 0.1% Tween 20). Rat serum was added and the plate was incubated for 1 hour at 37°C. After washing, horseradish peroxidase (HRP)-labeled goat anti-rat IgG (1∶2000, Jackson ImmunoResearch, West Grove, PA, USA) was applied and incubated for 1 hour at 37°C. After washing, 3, 3′, 5, 5′-tetramethylbenzidine (TMB) was added. The chromogenic reaction was terminated with 2 M H_2_SO_4_. Absorbance was measured at 450 nm with an ELISA reader (Multiskan MK3, Thermo Labsystems, Vantaa, Finland).

### Immunostaining

Following collection, rat and human kidney tissues were fixed in paraformaldehyde/lysine/periodate (PLP) solution for 2 hours followed by 18% sucrose overnight as previously described [30], [31]. Then, the tissues were embedded in Tissue-Tek OCT, snap-frozen in liquid nitrogen and cut using a freezing microtome (Thermo Cryotome E, Shandon, UK) to a thickness of 4 µm.

To observe IgG deposition in PHN rat glomeruli, cryosections were stained with fluorescein isothiocyanate (FITC)-conjugated goat anti-rat IgG (1∶50, Jackson ImmunoResearch, West Grove, PA, USA).

To observe the deposition of C5b-9 and podocyte injury in PHN rat glomeruli, cryosections were stained with mouse anti-rat C5b-9 (1∶200, Abcam, New Territories, Hong Kong) and mouse anti-rat desmin (1∶100, Abcam, New Territories, Hong Kong), followed by Alexa Fluor 594-conjugated goat anti-mouse IgG (1∶200, Jackson ImmunoResearch, West Grove, PA, USA).

To observe the expression of Angptl4 in glomeruli and its colocalization with podocytes, endothelium, mesangial cells, and GBM in PHN rats, cryosections were stained with goat anti-rat/human Angptl4 (1∶100, Santa Cruz Biotech, Delaware Avenue, CA, USA), mouse anti-rat synaptopodin (1∶10, Progen, Heidelberg, Germany), mouse anti-rat laminin (1∶400, Abcam, New Territories, Hong Kong), mouse anti-rat RECA-1 (1∶10, Abcam, New Territories, Hong Kong) and mouse anti-rat OX-7 (1∶200, Abcam, New Territories, Hong Kong), followed by Alexa Fluor 488-conjugated donkey anti-goat IgG (1∶200, Jackson ImmunoResearch, West Grove, PA, USA) and Alexa Fluor 594-conjugated donkey anti-mouse IgG (1∶200, Jackson ImmunoResearch, West Grove, PA, USA).

The procedure and antibodies for human kidney tissue immunostaining were the same as those used for rat kidney tissue immunostaining.

Confocal images were generated using an OLYMPUS FLUOVIEW FV1000 confocal microscope (Tokyo, Japan). All exposure settings were kept the same for each group of kidneys. Images were sequentially captured by digitally imaging the entire sagittal section, including the cortex and outer medulla (10–15 images). The fluorescence intensity was measured by manually outlining the perimeter of ten glomeruli in each section and semi-quantifying the luminosity of the outlined regions using image analysis software (ImageJ, version 1.47, National Institutes of Health, USA). A background correction was made for each glomerulus by subtracting the average intensity in non-stained regions (outlined manually) in the glomerulus. The colocalization ratio was analyzed with ImageJ using a colocalization plugin to calculate the colocalization area, which was then divided by the corresponding total area.

### Quantitative real-time PCR

The glomeruli of rats were isolated using the standard sieving method. RNA was isolated from the glomeruli using TRIzol Reagent (Invitrogen Life Technologies, Carlsbad, CA, USA). Total RNA was reverse transcribed using a high-capacity cDNA reverse transcription kit (Applied Biosystems, Foster City, CA, USA). The primer sequences for real-time PCR were as follows: Angptl4 primers 5′-ACGGCAAATGAGCTGGG-3′ and 5′-GGGCAGGGACAGGCCA-3′. For real-time PCR, 2×SYBR Green PCR Master Mix (Applied Biosystems, Warrington, UK) was used according to the manufacturer’s instructions with a Bio-Rad CFX96 Real-Time System (Singapore). There were 3 replicates for each sample. Experimental cycle threshold (CT) values were normalized to glyceraldehyde-3-phosphate dehydrogenase (GAPDH) measured on the same plate, and the fold differences in gene expression were determined using the 2^−ΔΔCT^ method [32].

### Western blot analysis of urinary and glomerular Angptl4

The glomeruli of rats were isolated using the sieving method. The glomeruli or the hepatic tissue was rinsed with ice cold 0.9% NaCl and lysed with lysis buffer (50 mM Tris, 150 mM NaCl, 1% sodium deoxycholate, 1% Triton X-100, 0.1% sodium dodecyl sulfate) containing protease and phosphatase inhibitors on ice. The lysates (100 µg) or centrifuged urine (20 µl) were denatured at 95°C for 5 min in sample buffer, separated in an 8% polyacrylamide sodium dodecyl sulfate gel, and transferred onto a PVDF membrane. The PVDF membranes were blocked at room temperature for 2 h with 5% powdered milk in Tris-HCl buffer containing 0.1% Tween 20 (TBST). Primary antibodies were diluted with TBST and added as follows: goat anti-rat Angptl4 (Santa Cruz Biotech, Delaware Avenue, CA, USA; 1∶200) and mouse anti-β-actin (Zsgb-Bio, Beijing, China; 1∶400). The membranes were incubated with primary antibodies overnight at 4°C followed by incubation with secondary antibodies (HRP-conjugated goat anti-mouse IgG and rabbit anti-goat IgG, Jackson ImmunoResearch, West Grove, PA, USA; 1∶5000) at room temperature for 1 h. There were 3 replicates for each sample. Blots were detected using a luminescent image analyzer (GE Healthcare Bio-Sciences AB, Uppsala, Sweden) and quantified using Image Quant TL (GE Healthcare Bio-Sciences AB, Uppsala, Sweden).

### Statistical analysis

All data are expressed as means ± standard deviations (SDs). Statistical analyses were conducted using one-way ANOVA with the LSD-t test, two-sample t test and Spearman’s coefficient of correlation analysis in SPSS (version 21.0). A value of p<0.05 was considered significant, and p<0.01 was considered highly statistically significant.

## Supporting Information

Figure S1 **Angptl4 expression in liver in passive Heymann nephritis (PHN) rats and normal rats. (A)** Western blot of Angptl4 in the liver of PHN rats on day 7 and normal rats. **(B)** Quantification of the western blot of Angptl4 expression in the liver. There was no significant difference between two groups (N = 8 for each group). **(C)** Reverse-transcription PCR of Angptl4 in the liver of PHN rats on day 7 and normal rats. **(D)** Quantification of reverse-transcription PCR of the Angptl4 expression in the liver. There was no significant difference between two groups (N = 8 for each group).
(TIF)

Figure S2 **Negative controls and normal control in passive Heymann nephritis (PHN) rats and diabetic nephropathy (DN). (A**) Negative control of PHN rats (magnification, x400). PHN, kidney tissue from PHN rats on day 7 stained with goat anti-rat Angptl4 antibody and the following secondary antibody; IgG negative control, kidney tissue from PHN rats on day 7 stained with goat IgG and the following secondary antibody; PBS negative control, kidney tissue from PHN rats on day 7 stained with PBS and the following secondary antibody. **(B**) Quantification of the immunofluorescence intensities of glomerular Angptl4 in (A) (N = 5 for each group). *P<0.01 VS. IgG negative control, PBS negative control and normal control. **(C**) Negative control of DN rats (magnification, x400). PHN, kidney tissue from DN rats on week 12 stained with goat anti-rat Angptl4 antibody and the following secondary antibody; IgG negative control, kidney tissue from DN rats on week 12 stained with goat IgG and the following secondary antibody; PBS negative control, kidney tissue from DN rats on week 12 stained with PBS and the following secondary antibody. **(D**) Quantification of the immunofluorescence intensities of glomerular Angptl4 in (C) (N = 5 for each group). *P<0.01 VS. IgG negative control, PBS negative control and normal control.
(TIF)
