# Supplementary figures and images for: The Calcineurin Inhibitor Tacrolimus Reduces Proteinuria in Membranous Nephropathy Accompanied by a Decrease in Angiopoietin-Like-4
Source: PLoS One. 2014 Aug 28;9(8):e106164. doi: 10.1371/journal.pone.0106164 (PMC4148427; doi:10.1371/journal.pone.0106164)

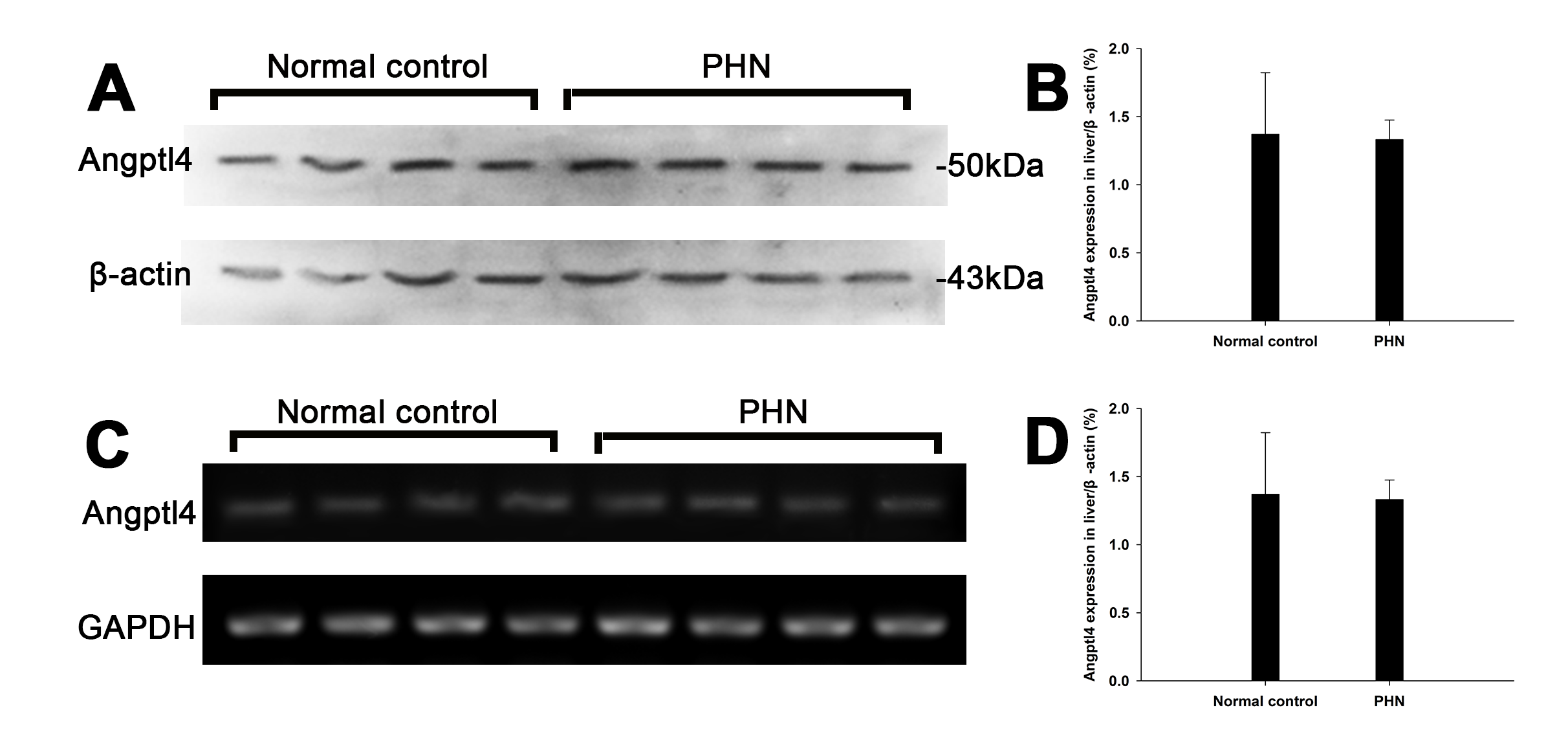

Supplement: Figure S1 — Angptl4 expression in liver in passive Heymann nephritis (PHN) rats and normal rats. (A) Western blot of Angptl4 in the liver of PHN rats on day 7 and normal rats. (B) Quantification of the western blot of Angptl4 expression in the liver. There was no significant difference between two groups (N = 8 for each group). (C) Reverse-transcription PCR of Angptl4 in the liver of PHN rats on day 7 and normal rats. (D) Quantification of reverse-transcription PCR of the Angptl4 expression in the liver. There was no significant difference between two groups (N = 8 for each group). (TIF) [file pone.0106164.s001.tif]

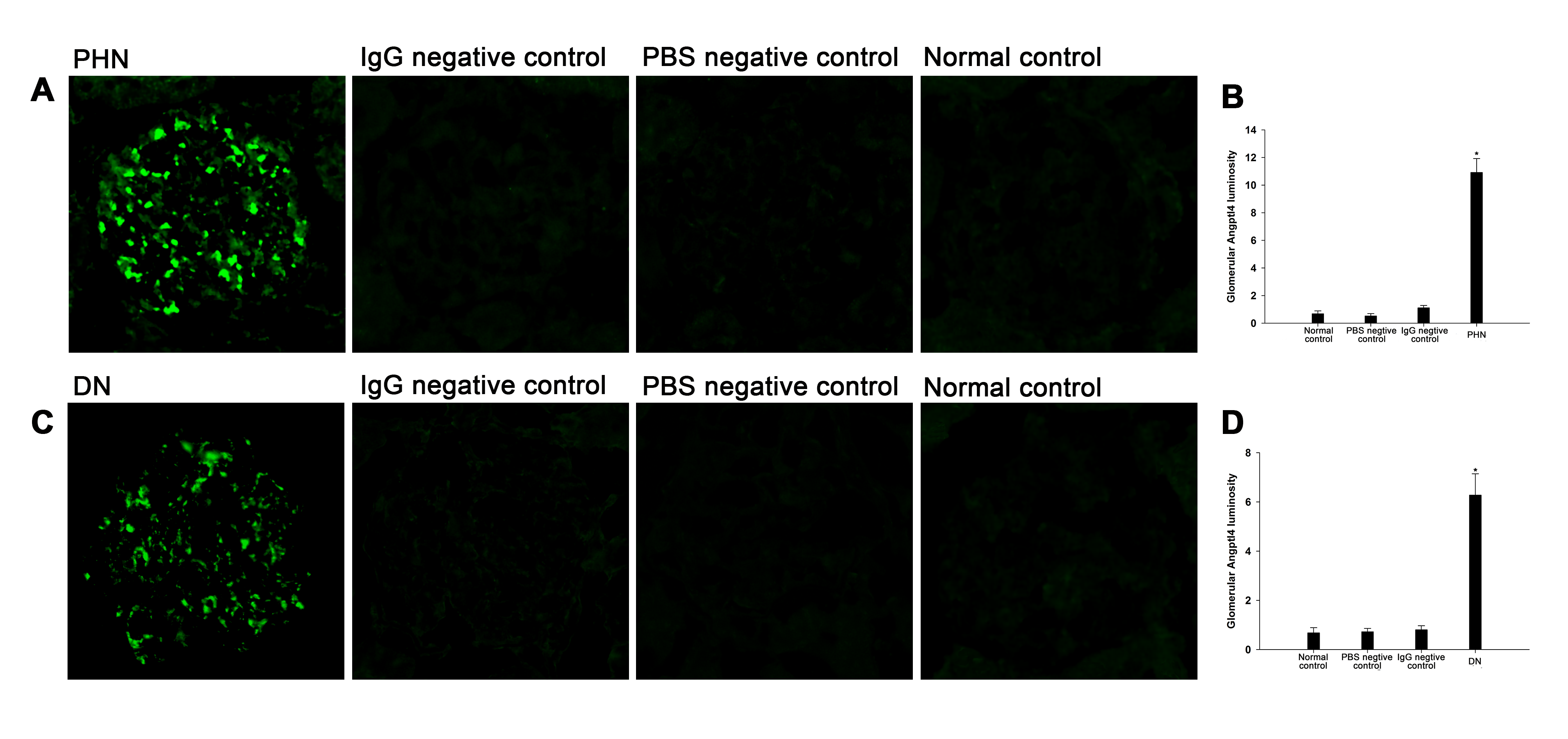

Supplement: Figure S2 — Negative controls and normal control in passive Heymann nephritis (PHN) rats and diabetic nephropathy (DN). (A) Negative control of PHN rats (magnification, x400). PHN, kidney tissue from PHN rats on day 7 stained with goat anti-rat Angptl4 antibody and the following secondary antibody; IgG negative control, kidney tissue from PHN rats on day 7 stained with goat IgG and the following secondary antibody; PBS negative control, kidney tissue from PHN rats on day 7 stained with PBS and the following secondary antibody. (B) Quantification of the immunofluorescence intensities of glomerular Angptl4 in (A) (N = 5 for each group). *P<0.01 VS. IgG negative control, PBS negative control and normal control. (C) Negative control of DN rats (magnification, x400). PHN, kidney tissue from DN rats on week 12 stained with goat anti-rat Angptl4 antibody and the following secondary antibody; IgG negative control, kidney tissue from DN rats on week 12 stained with goat IgG and the following secondary antibody; PBS negative control, kidney tissue from DN rats on week 12 stained with PBS and the following secondary antibody. (D) Quantification of the immunofluorescence intensities of glomerular Angptl4 in (C) (N = 5 for each group). *P<0.01 VS. IgG negative control, PBS negative control and normal control. (TIF) [file pone.0106164.s002.tif]
